# A Cohort Study Characterizing the Outcomes Following an Acute SARS-CoV-2 Infection in Pregnancy

**DOI:** 10.3390/jcm14217869

**Published:** 2025-11-06

**Authors:** Clementine Adeyemi, Leticia Breuer, Raghad Kodvawala, Delia Miller, Margaret V. Powers-Fletcher

**Affiliations:** 1 Department of Internal Medicine, College of Medicine, University of Cincinnati, 3230 Eden Ave, Cincinnati, OH 45267, USA; adeyemce@mail.uc.edu; 2 College of Medicine, University of Cincinnati, 3230 Eden Ave, Cincinnati, OH 45267, USA; 3 Department of Environmental and Public Health Sciences, University of Cincinnati, 160 Panzeca Way, Cincinnati, OH 45267, USA; mille5dp@ucmail.uc.edu; 4 College of Arts & Sciences, University of Cincinnati, 2700 Campus Way, Cincinnati, OH 45219, USA

**Keywords:** COVID-19 and pregnancy, SARS-CoV-2 infection, maternal morbidity, pregnancy complication, management, maternal comorbidities, severe disease, prevention, diagnosis, treatment

## Abstract

**Background/Objectives:** Current estimates suggest that 6% of COVID-19 survivors develop a post-viral sequela known as Long COVID. Among those at risk for this sequela, pregnant individuals are a vulnerable patient population, but they are understudied as to the nature of their symptomology and potential adverse outcomes. **Methods:** This retrospective study evaluated a cohort of 150 pregnant individuals with a history of acute SARS-CoV-2 infection during pregnancy, observing for Long COVID symptoms and assessing for adverse outcomes. Of this cohort, 64% identified as Black and/or Latina, which provides a more diverse representation compared to previously published studies. **Results:** Within this cohort, 26.7% of individuals experienced at least one symptom of Long COVID; subcohorts, which were categorized based on presence or absence of Long COVID symptomology, presented with varying phenotypes. Pain, mental health dysfunction or psychological problems, and fatigue were the predominant symptoms documented for patients who averaged two Long COVID symptoms after at least 30 days following a COVID-19 diagnosis. Different adverse outcomes were higher in frequency among subcohorts, highlighting a need for continued study to explore the nuances of the impact of COVID-19 on this unique and vulnerable population. The most notable trends between subcohorts related to treatment patterns for acute COVID-19, vaccine status, and cesarean delivery rates. **Conclusions:** By providing a description of the documented health experience for a predominantly non-White cohort of individuals who were diagnosed with an acute SARS-CoV-2 during pregnancy, our study contributes to a foundation upon which future studies can build.

## 1. Introduction

First described through social media by sufferers who characterized themselves as “long-haulers,” the presence or continuation of symptoms for an extended period following acute COVID-19 is an accepted condition with various working definitions [1]. The 2022 definition by the Centers for Disease Control and Prevention (CDC) defined it as “a lack of return to normal health after a SARS-CoV-2 infection, specifically after at least four weeks,” while the National Academies of Sciences, Engineering, and Medicine (NASEM) defines it as “an infection-associated chronic condition (IACC) that occurs after a SARS-CoV-2 infection and is present for at least 3 months as a continuous, relapsing and remitting, or progressive disease state that affects one or more organ systems” [2,3]. The phrase “Post-Acute Sequelae of SARS-CoV-2 infection,” or PASC, is used by the National Institutes of Health (NIH) to refer to the worsening, continuation, reoccurrence of symptoms or the emergence of new symptoms 30 days or more after the initial manifestation of symptoms from a SARS-CoV-2 infection, while the World Health Organization (WHO) definition is specific to symptom continuation of three months or longer [4,5]. Using the WHO definition, at least 6% of the over 778 million cases with a history of COVID-19 globally are thought to have experienced Long COVID and, in the United States specifically, this condition afflicts an estimated ~43–45 million people [5,6,7,8]. Due to the variability in clinical definitions and challenges in diagnosis, however, this number is likely underestimated [9].

Not only does the variability within clinical definitions of Long COVID have an impact on epidemiology estimates, but it also has a negative impact on potential sufferers, delaying them from seeking care due to the lack of knowledge on the comprehensive or differential symptoms; this is amplified by healthcare access challenges such as frequently fragmented, siloed care across various clinical specialties [10,11]. This is particularly concerning for vulnerable patient populations who may be at higher risk for adverse outcomes. Researchers are working to better characterize Long COVID in such vulnerable groups [12,13,14,15], but there are still critical gaps in knowledge.

Pregnant individuals are within the group of particularly vulnerable patients for whom a better understanding of Long COVID is required. Adverse effects of acute COVID-19 have been documented during pregnancy, including higher risk of pre-eclampsia and increased incidence of neurodevelopmental disorders in offspring [16,17,18]. Moreover, because of safety concerns for the fetus, clinical trials and subsequently approved therapies for acute COVID-19 in pregnant individuals are limited [19,20,21]. Overall, there is a higher risk of respiratory-infection-associated maternal and fetal morbidity and mortality in pregnant individuals, including associated conditions such as influenza [22,23,24]. The exact nature of Long COVID in pregnant or recently pregnant individuals, however, is still poorly understood and is possibly unique from that of non-pregnant individuals. Pregnant individuals are known to have physiologically altered immune states during pregnancy and post-partum compared to non-pregnant individuals, among other physiological differences [25]. As a result, variation in susceptibility to and presentation of disease is not surprising [26]. Other diseases have been documented as presenting with distinct characteristics in pregnant individuals compared to non-pregnant individuals, including diabetes and hypertension [27,28,29]. Long COVID in the pregnant and post-partum population is still poorly understood, with the few published studies reporting conflicting conclusions [14,30,31,32]. Thus, to add to the emerging body of research and literature, this study aims to describe the health status of a cohort of individuals who experienced acute SARS-CoV-2 infection during pregnancy. This cohort was selected from those seeking care at an academic health center in an urban setting, which serves both rural and suburban areas. As a result, this study, with its comparatively diverse patient population, offers a distinct perspective from existing studies on Long COVID during pregnancy [14,15].

## 2. Methods

**Figure 1:**
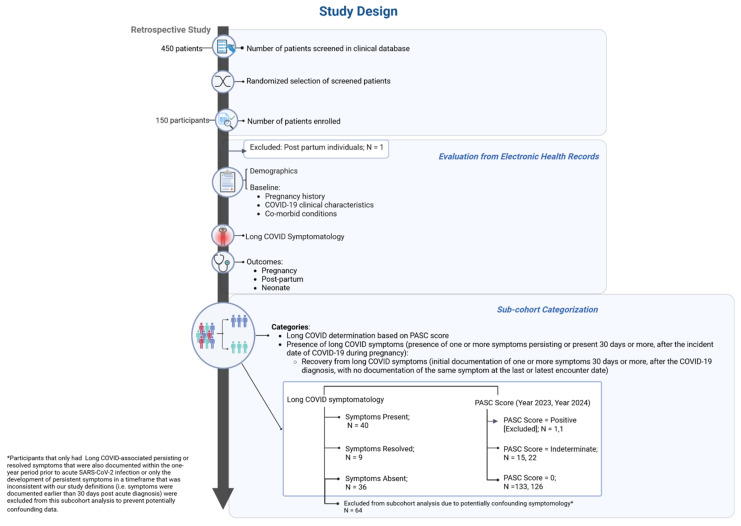
Study Design. In this descriptive study, electronic health records were retrospectively reviewed for 149 pregnant individuals with a history of acute SARS-CoV-2 infection during pregnancy, observing for Long COVID symptoms and assessing for adverse outcomes. Participants were classified according to two different classification systems, either based on overall Long COVID symptomatology or a PASC score derived from the NIH RECOVER protocol. Figure created using the web-based application, BioRender. Adeyemi, C. (2025) https://BioRender.com/g0xjvir (accessed on 30 September 2025).

This study was a retrospective cohort study conducted in May through December of 2024 involving the chart review and unstructured data analysis of the electronic health records (EHRs) of pregnant individuals seen within a singular large academic healthcare system that consists of two regional hospitals and multiple clinical sites (Figure 1).

### 2.1. Human Subjects Protection

This study was conducted with the review and approval of the University of Cincinnati Institutional Review Board (protocol # 2020-0313).

### 2.2. Cohort Selection and Definitions

A cohort of 150 participants, aged 15 years and above, was randomly selected from a pre-existing list of 450 patients that was generated by network providers to track pregnancy-related encounters, particularly labor and delivery, for patients with documented SARS-CoV-2 infections at any point during pregnancy. Pregnancy was noted by a clinician’s observation of any gestational age for the patient and an indicator of pregnancy such as a prenatal visit, ultrasound visit, a delivery summary note or, a post-partum encounter detailing the date of delivery. Infection was documented by the clinician within patient’s EHR based on either an in-house laboratory-confirmed positive SARS-CoV-2 PCR or antigen test or an at-home test result noted by the patient. The acute phase of a SARS-CoV-2 infection was defined as from the date of reported symptom onset up to 21 days. Upon EHR review, a single patient was identified who had a post-partum COVID-19 diagnosis date and was thus excluded from the study, resulting in a total of 149 participants.

### 2.3. Study Period

Participants records were reviewed from a year prior to their pregnancy-related COVID-19 diagnosis date until 31 December 2024.

### 2.4. EHR Review

Data collection was performed manually by a team of four researchers (C.A, R.K, L.B and D.M) using a data abstraction tool that was informed by expertise from a panel of individuals with maternal–fetal medicine, Long COVID, and/or EHR informatics expertise. Each investigator was trained using a protocol detailing search terms to use for each question on the two instruments used for this study (Supplemental Figure S1). These instruments evaluating symptomology and pregnancy were adapted for EHR data extraction from the NIH RECOVER instruments for the prospective study conducted on pregnant adults (Table 1). This adapted instrument included select symptomology associated with PASC along with variables associated with pregnancy history and outcomes.

### 2.5. Data Collection Variables

From the manual EHR review, clinical chart documentation of 26 selected symptoms that have previously been associated with Long COVID was recorded as outlined in Table 1. During the period of review, several time points were specifically assessed for symptom documentation, including the following windows relative to the COVID-19 diagnosis index date during pregnancy: a year before, 22 days after, 30 days after, and the final encounter at the time of record review. Participant demographic data included age (at the time of expected delivery date), race, and ethnicity, as well as SARS-CoV-2 vaccine status. In addition to symptom documentation, data was collected related to health during pregnancy and delivery outcomes. Included in the outcomes evaluated was COVID-19 severity indicated by a documentation of the need for ventilation or hospitalization due to acute COVID-19. Participants with missing data were retained for analysis to avoid a significant loss of information and a reduction in sample size.

### 2.6. Cohort Classification

For analysis of symptoms following COVID-19 diagnosis during pregnancy, participants were classified using two different approaches: (1) by overall symptom presence, recovery, or absence and (2) based on PASC score derived from the NIH RECOVER protocol (Figure 1).

#### 2.6.1. Overall Symptom Presence

Long COVID symptomology was classified as “Symptoms Present,” “Symptoms Resolved,” or “Symptoms Absent.” For those classified as Symptoms Present, our study design required that the symptom be present at both 30 days or more following the acute COVID-19 and remain documented at the final encounter in the study period; if the symptom was no longer documented at the final encounter, the participant was classified as Symptoms Resolved. Participants who did not clearly meet the criteria for one of these three classifications were excluded from analysis as outlined in Figure 1. Additionally, Supplemental Table S1 lists each participant record by their subcohort classification group.

#### 2.6.2. PASC Score

The NIH RECOVER group developed an index scoring model for the description of patients suspected of or at-risk for PASC [4]. Briefly, the scoring was informed by the most distinct frequent symptoms experienced by their Long COVID participants compared to those who did not have Long COVID and those who did not have a positive case of SARS-CoV-2 infection. The cohort was an adult population of predominantly non-pregnant individuals. The initial index was published in 2023 with an updated 2024 index informed by the evolving knowledge of the Long COVID experience informed by their participants [33]. The categories are “PASC Zero”, “PASC Indeterminate.” or “PASC Positive.” PASC Positive participants have a score of 12 or more in 2023 and 11 in 2024 with these scores determined from the aggregate score of the select symptoms reported or observed in participants.

For the purpose of our study, participants were classified as “PASC Positive” if they had documented persistence or presence of one or more PASC-specific symptoms, culminating in an aggregate score of 11 or 12, depending on the version of the PASC scoring system used that year. Participants were considered “PASC Indeterminate” if they had documented persistence or presence of one or more PASC symptoms with an aggregate score of less than 11 or 12, depending on the PASC score version used. Finally, participants were considered “PASC Zero” if they either: (1) only had a documented presence of one or more symptoms that were also present at some point during the year before the incident date of their COVID-19 diagnosis during pregnancy; (2) only had a documented presence of one or more symptoms persisting or present 30 days or more after their incident date of COVID-19 during pregnancy but a final PASC score of zero; (3) only had a documented presence of one or more symptoms present 30 days or more after their incident date of COVID-19 diagnosis during pregnancy but no longer had documentation of that symptom in their latest encounter at the time of chart review; or (4) had no documentation of any of the PASC score symptoms in their EHR.

### 2.7. Statistical Analysis

The sample size was determined to balance the intensive effort required for comprehensive manual review of each participant’s electronic health record with the goal of assembling a preliminary yet informative cohort representative of this understudied population. Because the study was exploratory and descriptive, it was not designed for hypothesis testing or formal power analysis. Descriptive statistics are presented as frequencies, proportions and means, as appropriate. For categorical variables highlighted within the text and figures, we reported both observed proportions and corresponding 95% confidence intervals to convey the precision of the estimates. Given the relatively small subgroup sizes (approximately 20–40 participants), exact (Clopper–Pearson) binomial confidence intervals were used rather than large-sample approximations.

## 3. Results

### 3.1. Subcohorts Based on Overall Symptom Presence and PASC Score Classification

#### 3.1.1. Overall Symptom Presence

**Figure 2:**
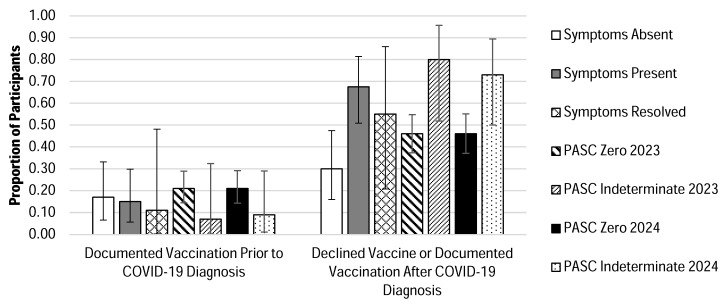
Observed Proportions of Vaccination History. For each subgroup, the observed proportions of participants for whom (1) COVID-19 vaccination was documented prior to the acute COVID-19 infection during pregnancy or (2) COVID-19 vaccination was either documented as declined by the participant or only documented after the acute COVID-19 diagnosis are reported. Corresponding 95% confidence intervals are shown to convey the precision of the estimates. The order of the chart legend entries (listed vertically) corresponds to the order of data categories shown in the graph (left to right).

There were 36 (24%), 40 (27%), and 9 (6%) of the total participants that were classified as Symptoms Absent, Symptoms Present, and Symptoms Resolved, respectively (Figure 1 and Table 2). The remaining 43% of participants could not clearly be classified into one of these three groups because of either the presence of Long COVID-associated symptoms prior to acute SARS-CoV-2 infection or the development of persistent symptoms in a timeframe that was inconsistent with our study definitions (Figure 1 and Supplemental Table S1); they were excluded from analysis, rather than included in one of the three classifications, so as to prevent potentially confounding data. Demographic data for subcohorts are listed in Table 2. For each subcohort, the majority of participants were in their third trimester at the time of acute COVID-19 diagnosis (Symptoms Absent 69%, Symptoms Present 55% and Symptoms Resolved 78%). Only 30% of participants in the Symptoms Absent subcohort had documentation of either declined SARS-CoV-2 vaccination or vaccination occurring after their acute COVID-19 diagnosis during pregnancy, compared to 67.5% in the Symptoms Present and 55% in the Symptoms Resolved subcohorts (Table 2 and Figure 2).

#### 3.1.2. PASC Score

The PASC Score classification was completed using models derived from those published in 2023 and 2024 [4,33], and thus subcohorts are labeled as such. Using the model adapted from the 2023 publication, there were 133 (89%) of the total participants that were classified as PASC Zero compared to 15 (10%) classified as PASC Indeterminate. Using the model adapted from the 2024 publication, there were 126 (85%) of the total participants that were classified as PASC Zero compared to 22 (15%) classified as PASC Indeterminate and one individual classified as PASC Positive (Figure 1). Demographic data for subcohorts are listed in Table 2. The majority of participants in all subcohorts were in their third trimester at the time of COVID-19 diagnosis. For the 2023 PASC Zero subcohort, only 46% of participants declined SARS-CoV-2 vaccination or were not vaccinated until after their COVID-19 diagnosis compared to 80% for the 2023 PASC Indeterminate subcohort. Similarly, for the 2024 PASC Zero subcohort, only 46% of participants declined vaccination or were not vaccinated until after their COVID-19 diagnosis during pregnancy compared to 73% in the PASC Indeterminate subcohort (Figure 2). Based on the model adapted from the 2023 publication and 2024 publication, the same individual was classified as PASC Positive with a vaccine status of declined vaccination.

### 3.2. Documentation of Symptoms Prior to Acute COVID-19 Diagnosis

Eighteen participants (11%) out of the entire cohort had documentation of at least one of the 26 evaluated symptoms persisting 30 days or more following their COVID-19 diagnosis during pregnancy but also had that same symptom documented in their EHR up to a year before their COVID-19 diagnosis. Because of this previous documentation prior to their acute COVID-19 during pregnancy, these occurrences were not considered “persistent symptoms” and thus, did not result in classification within the Long COVID Present or PASC Indeterminate subcohorts; participants would have needed documentation of at least one other persisting symptom that was not present within the year prior to COVID-19 to be classified within either of these groups.

### 3.3. Symptoms Following Acute SARS-CoV-2 Infection During Pregnancy

**Figure 3:**
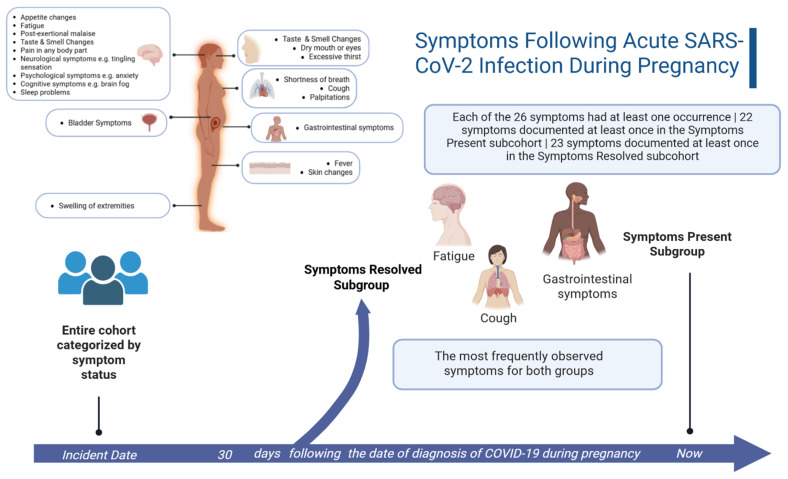
Symptoms Following Acute SARS-CoV-2 Infection During Pregnancy. Participants were classified based on overall Long COVID symptomatology based on a retrospective EHR analysis for observed documentation of 26 different symptoms. For those classified as Symptoms Present, our study design required that the symptom be present at both 30 days or morefollowing the acute COVID-19 and remain documented at the final encounter in the study period; if the symptom was no longer documented at the final encounter, the participant was classified as Symptoms Resolved. Figure created using the web-based application, BioRender. Adeyemi, C. (2025) https://BioRender.com/r8gb1n6 (accessed on 30 September 2025).

Of the Long COVID symptoms documented as persisting until the latest encounter within the study window, fatigue (15%), cough (13%) and gastrointestinal symptoms (10%) are the most frequently observed symptoms seen in the 40 participants categorized in the subcohort with Symptoms Present (Figure 3): The most frequently documented symptoms that were present at least 30 days after a COVID-19 diagnosis but not persisting till the latest encounter were similar to those that persisted, with the exception of frequent documentation of resolved pain anywhere in the body. Overall, however, the majority of participants did not have documentation of many of the Long COVID symptoms that were evaluated (Figure 3). For example, 60% of the participants did not have any documentation of pain following their acute COVID-19 diagnosis and there was no recorded documentation of color changes in the skin for 99% of participants. Each of the 26 symptoms were documented as occurring following the COVID-19 diagnosis in at least one participant, however (Table 1 and Figure 3). Twenty-two of the twenty-six symptoms are documented as persisting for at least one participant, while twenty-three symptoms were documented for at least one participant, as resolving at least 30 days or more following the incident date of a SARS-CoV-2 infection during pregnancy.

### 3.4. Pregnancy and Neonatal Outcomes

#### 3.4.1. Outcomes Based on Overall Symptom Presence Classification

**Figure 4:**
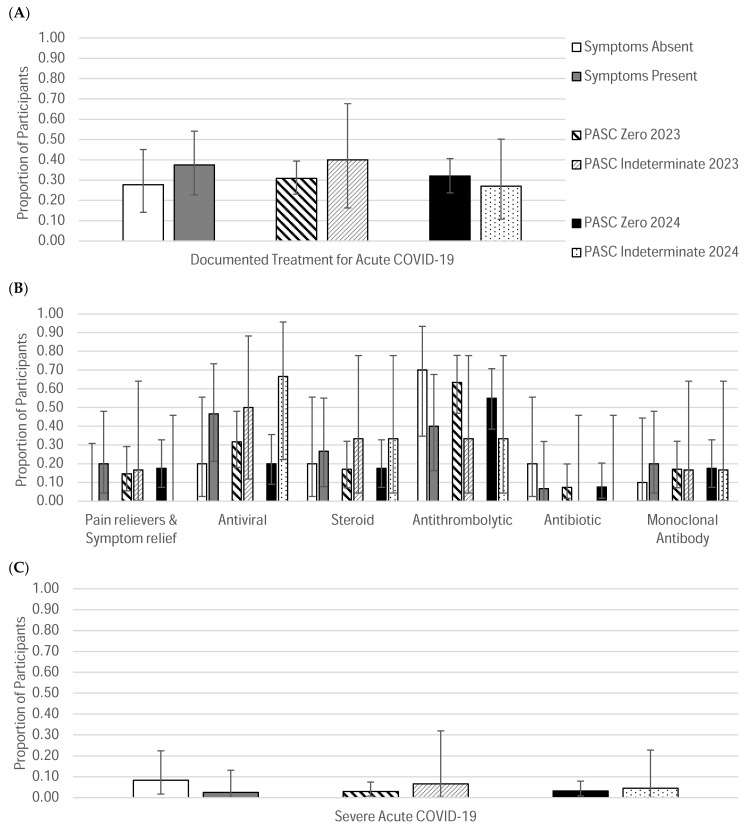
Observed Proportions of Acute COVID-19 Treatment History and Severity. (**A**) For each subgroup, the observed proportions of participants for whom treatment for acute COVID-19 was documented are reported. (**B**) Of those who had documented treatment, the observed proportion of the different treatment options given is also reported. (**C**) For each subgroup, the observed proportions of severe acute COVID-19 are reported. Corresponding 95% confidence intervals are shown to convey the precision of the estimates. The chart legend provided in panel A corresponds to all three panels. The order of the chart legend entries (listed vertically) corresponds to the order of data categories shown in the graph (left to right).

**Figure 5:**
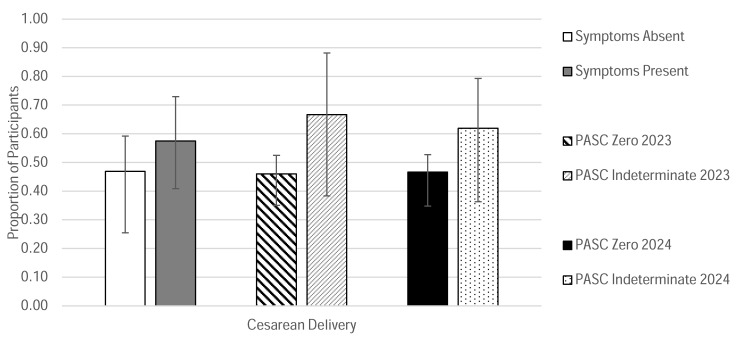
Observed Proportions of Cesarean Delivery. For each subgroup, the observed proportions of participants who underwent cesarean delivery are reported. Corresponding 95% confidence intervals are shown to convey the precision of the estimates. The order of the chart legend entries (listed vertically) corresponds to the order of data categories shown in the graph (left to right).

The Symptoms Absent subcohort had a greater proportion of participants for whom the incident pregnancy was their first pregnancy (19% vs. 5% for the Symptoms Present subcohort). Antithrombolytics were the most frequently prescribed medication during acute SARS-CoV-2 infection in the Symptoms Absent subcohort, whereas antivirals had been the most commonly prescribed medications in the Symptoms Present subcohort (Figure 4B and Table 3). More severe COVID-19 cases were noted in the Symptoms Absent group (8.3% in the Symptoms Absent group vs. 2.25% in the Symptoms Present group) (Figure 4C and Table 3). Both groups had relatively high rates of cesarean delivery (CS) compared to national rates; most of the cesarean deliveries were planned [34]. Cesarean deliveries trended higher in the Symptoms Present subcohort (58%) compared to the Symptoms Absent subcohort (47%). Both subcohorts had documented comorbidities (Table 3 and Figure 5).

#### 3.4.2. Outcomes Based on PASC Score Classification

The most frequently prescribed medication during acute SARS-CoV-2 infections for the 2023 and 2024 PASC Zero subcohorts were antithrombolytics (63% and 55%, respectively). Antivirals were the most prescribed medication for the 2023 and 2024 PASC Indeterminate subcohorts (50% and 67%, respectively) (Figure 4). Different adverse outcomes were documented in greater rates for each of the subcohorts, without a specific trend observed (Table 4 and Figure 5).

## 4. Discussion and Conclusions

This descriptive retrospective cohort study examined the experiences of individuals diagnosed with acute COVID-19 during pregnancy, as documented in electronic health records, to advance understanding of Long COVID phenotypes in vulnerable populations. Using a manual EHR review approach encompassing clinical encounters up to one year prior to and following acute COVID-19 diagnosis, the EHRs of 149 participants that were randomly selected from an at-risk patient list at a single healthcare network serving the tri-state region of Ohio, Kentucky, and Indiana were evaluated. Participants were classified into subcohorts based on the persistence of symptoms associated with Long COVID or an adaptation of the published PASC scoring models; these two different cohort classifications were applied to reduce the risk of bias introduced by using a single classification system for a condition in an understudied population. Data was analyzed for trends related to symptom documentation and pregnancy-related outcomes to provide additional insight and an expanded scope of understanding of the complications that may occur following an acute SARS-CoV-2 infection in a pregnant individual.

A key strength of our study was the demographic diversity of the patient population. Unlike many previously published reports, our study cohort is predominantly made up of individuals who identify as Black and/or Latina. As such, it offers a perspective that is representative of the most vulnerable patient populations within the context of unacceptably high maternal morbidity and mortality rates in the United States, accounted for by racialized pregnant individuals intersecting with the disproportionate morbidity and mortality from COVID-19 reported in several racialized groups [34,35].

The documentation of persistence of at least one of the 26 symptoms previously associated with Long COVID was observed for 26.7% of the cohort. For a symptom to be considered persistent, our study design required that it be present at both 30 days or more following the acute COVID-19 and remain documented at the final encounter in the study period. This definition likely results in a conservative measure for evaluating Long COVID symptomology given the risk of both variability in symptom presentation throughout time [3], as well as bias introduced by the limitation of using symptoms documented in the EHR alone. An additional 6% of the cohort were classified as Symptoms Resolved in our study because their symptom(s) were no longer documented at the time of the final encounter. Despite the conservative nature of our estimate, however, our observed rate is higher than that reported in other reports [14,15]. This may be due to a resolution of symptoms after the 30-day time point as the other studies reported at three months or longer following a COVID-19 diagnosis in pregnancy. In contrast, other studies have reported a higher rate of up to 35% of Long COVID during pregnancy [14,15,30,31].

In our subcohort of participants experiencing persistent symptoms following acute infection, the majority of participants (78%) in the Symptoms Present subcohort had only one persisting symptom. The most frequent symptoms documented in our Symptoms Present and PASC Indeterminate subcohorts mirrored the predominant symptoms seen in adult sufferers of Long COVID, including pain and psychological symptoms [4]. Relying on EHR documentation alone, the nuances of the pain and psychological symptoms experienced are unclear. It may be interpreted as manifestation caused by the pathophysiology of Long COVID itself, or may be a reflection of the harm and the reduction in the quality of life experienced by patients who are seeking adequate care in the context of an emerging and poorly understood chronic disease [36,37,38]. Further study is required to gain additional qualitative insight directly from patients themselves, which would provide the necessary context for the interpretation of this finding.

The symptoms least documented in the EHR included skin color change and difficulty getting pregnant, the latter of which is explained by the fact that the study cohort was pregnant or post-partum. Skin color change may not be as apparent in individuals that are highly melanated and, thus, is perhaps a less informative symptom for the diagnosis of Long COVID in a non-White patient population. Surprisingly, one infrequently reported symptom was a loss or change in one’s sense of taste or smell. The loss of these two senses is a distinct symptom of acute COVID-19 and the second highest scored symptom within the PASC determination score [4,33]. Pregnancy itself has been known to come with a change in one’s sense of taste and smell [39], however, and this may have had an impact on either patient reporting or provider documentation of this symptom within the EHR.

The most notable trends between subcohorts were related to treatment patterns for acute COVID-19, vaccine status, and cesarean delivery rates. Participants in the Symptoms Absent subcohort had less frequently received treatment for their SARS-CoV-2 infections compared to those in the Symptoms Present group. Additionally, a higher proportion of participants in either the Symptoms Present, Symptoms Resolved, or PASC Indeterminate subcohorts had either declined SARS-CoV-2 vaccination or had received their vaccine following their COVID-19 diagnosis during pregnancy compared to the Long COVID Absent or PASC Zero cohorts. This finding suggests a connection between vaccination status and persistence of symptoms following acute infection in pregnant individuals. Although no inferential statistical analyses were performed in this descriptive study, including adjustments for potential confounders such as comorbidities or access to care, this observation is consistent with previous reports in the literature that support vaccination as an effective approach for reducing the risk of Long COVID [40]. Finally, overall, cesarean deliveries were unusually high in our cohort compared to national rates [41]. It is well-documented, however, that pregnant individuals of color tend to have higher rates of cesarean delivery in the United States compared to White individuals, which is a disparity that is not only a significant public health concern but also may introduce a confounding variable when evaluating the impact of Long COVID on labor and delivery outcomes in our cohort [41]. Furthermore, there are known adverse outcomes attributed to cesarean delivery, which can further confound our analysis due to the high rates observed in all cohorts, even those without persistent symptoms.

Long COVID in pregnancy is still relatively understudied. At the time of submission, this is only one of a few publications on the persistence of symptoms following acute COVID-19 during pregnancy in the United States. As such, the reported work provides important contributions to our understanding of human health, particularly because of a number of strengths in our approach. First, rather than relying on diagnosis codes or insurance claims data to identify participants, we manually reviewed EHR data, including unstructured clinical notes, to classify participants. Due to the risk of underdiagnosis or misdiagnosis of Long COVID as an emerging condition over the past five years, reliance on diagnosis codes or claims data alone would likely misidentify or fail to capture potentially undiagnosed but symptomatic Long COVID cases [11]. Of note, only 2 of the 149 patients in our cohort had any documentation of a Long COVID diagnosis in their EHR. Additionally, for further thoroughness, our manual review included analysis of symptoms up to a year before the diagnosis of COVID-19 during pregnancy and persistence of symptoms as early as three weeks following an acute SARS-CoV-2 infection. These windows of time provided us with the broadest approach to identifying symptoms that may have been present before the concurrent conditions of pregnancy and acute COVID-19, as well as allowed for a more inclusive dataset regarding persistence of symptoms.

To our knowledge, this is the first study to apply the previously published PASC scoring algorithm to an independent dataset, specifically within a pregnant population. As such, this cohort study provides important proof-of-principle evidence related to the feasibility and potential implications of such application. To date, there have been two versions of a PASC scoring algorithm published [4,33]. There have been other studies utilizing these algorithms, but they have all been within the NIH RECOVER team or do not include a pregnant population [4,18,33,42]. A limitation with the application of the scoring algorithm based on evaluation of health records alone was the inability to score certain topics that may not routinely be discussed or documented in the EHR. This included documentation regarding sexual desire, abortion, and fertility. Other symptoms might not be documented due to the area of specialty such as teeth loss. Another concern is temporality, as participants varied in the frequency of their encounters with clinicians within the healthcare system.

Limitations of our study overall include those that are well-established as being associated with any study that utilizes a retrospective EHR review [43]. EHR documentation does not reflect the entirety of a patient’s health experience, particularly as the documentation is conducted by a clinician and thus, includes predominantly clinician-observed outcomes, as opposed to direct patient reported outcomes or experiences. There is also a likelihood of observer bias in the reporting of the patient’s experience. Additionally, data in EHRs does not always reflect the real time experiences of the patient, in that patients do not necessarily interact with their healthcare provider every time they have a health-related incident. Underserved patient populations who have reduced healthcare access and fewer encounters with clinicians may also have less EHR documentation. As our study cohort is predominantly made up of individuals who may fall within these underserved patient populations, this bias may influence our ability to fully capture the lived experience of this cohort and this is an inherent limitation to retrospective EHR review. Further work to authentically capture and describe the lived experience of pregnant individuals who are at risk for Long COVID, particularly those from underserved communities, is critical to build upon the foundation of our descriptive study reported here. Given the limited awareness of Long COVID in many vulnerable patient populations, evaluating the Long COVID experience in pregnant individuals from their direct report has the additional benefit of increased community awareness, which can ultimately contribute to a reduction in stigma, an increase in healthcare seeking behaviors, and combat underdiagnosis [44,45]. Additionally, expanding this work to other sites and including additional variables such as socioeconomic data is critical to enhancing the generalizability of these findings. Our study contributes to a foundation upon which these future studies can build, however, by providing a description of the documented health experience for a predominantly non-White cohort of individuals who were diagnosed with an acute SARS-CoV-2 during pregnancy. The trends observed in potential risk factors and health outcomes based on presence or absence of Long COVID symptoms serve to inform future clinical studies and qualitative research designed to further delve into the nuances of Long COVID in vulnerable patient populations.

## Figures and Tables

**Table 1:** List of Symptoms Evaluated through Retrospective EHR Chart Review.

Symptom List/Description
Poor appetite or overeating
Fatigue (being very tired)
Post-exertional malaise (Symptoms worse after even minor physical or mental effort)
Swelling of legs, Weakness in arms or legs, muscle cramps in legs and/or feet
Fever, chills, sweats or flushing
Loss of or change in smell or taste
Pain in any part of your body
Shortness of breath
Cough
Palpitations, racing heart, arrhythmia, skipped beats
Gastrointestinal (belly) symptoms (feeling full or vomiting after eating, diarrhea, constipation, cramping or colicky abdominal pain)
Bladder problems (incontinence, trouble passing urine or emptying bladder)
Nerve problems (tremor, shaking, abnormal movements, numbness, tingling, burning, cannot move part of body, new seizures)
Problems with anxiety, depression, stress, or trauma-related symptoms like nightmares or grief
Problems with sleep
Problems thinking or concentrating (“brain fog”), Feeling faint, dizzy, “goofy”; difficulty thinking soon after standing up from a sitting or lying position
Color changes in your skin, such as red, white or purple
Skin rash, sores
Excessively dry mouth, eyes
Excessive thirst
Vision problems (blurry, light sensitivity, difficulty reading or focusing, floaters, flashing lights, “snow”)
Problems with hearing (hearing loss, ringing in ears)
Hair loss
Problems with teeth
Changes to menstrual cycle, reports of heavy periods
Changes in fertility or difficulty getting pregnant
Any other symptoms that were attributed to COVID-19 in EHR documentation

**Table 2:** Subcohorts Based on Overall Symptoms and PASC Score.

		Symptoms Absent	Symptoms Present	Symptoms Resolved	PASC Zero 2023	PASC Indeterminate 2023	PASC Zero 2024	PASC Indeterminate 2024
Number of participants		36	40	9	133	15	126	22
Proportion of participant pool		24.20%	26.90%	6.04%	89.30%	10.10%	84.60%	14.80%
Age (years)	Range	16–41	20–44	17–36	15–41	20–39	16–41	15–39
	Mean	26.6	32.4	26.7	27.7	28.9	28.1	27.1
Race/Ethnicity	Black	31%	47.50%	22%	43%	47%	44%	36%
	White	32%	30%	22%	30%	33%	29%	41%
	Latina	32%	17.50%	56%	22%	13%	22%	14%
	Asian	0%	5%	0%	2%	7%	2%	5%
	Native American or Alaska Native	0%	0%	0%	1%	0%	0%	5%
	Native Hawaiian or Pacific Islander	0%	0%	0%	0%	0%	0%	5%
	Multiracial	5%	0%	0%	2%	0%	2%	0%
Vaccination Status	Before COVID-19 diagnosis	17%	15%	11%	21%	7%	21%	9%
	After COVID-19 diagnosis	19%	27.50%	33%	20%	40%	21%	32%
	Not documented	53%	17.50%	33%	33%	13%	33%	18%
	Patient declined	11%	40%	22%	26%	40%	25%	41%
Trimester on date of COVID-19 Diagnosis	1st	3%	12.50%	0%	5%	20%	5%	14%
	2nd	28%	32.50%	22%	32%	47%	35%	50%
	3rd	69%	55%	78%	62%	33%	60%	36%
Gestational Age (weeks)	Mean	31.9	28	33.6	29	25.7	29.5	25.6
Trimester	Mean	3rd	3rd	3rd	3rd	2nd	3rd	2nd

**Table 3:** Characteristics and Outcomes Associated with Pregnancy Based on Presence or Absence of Symptoms.

		Symptoms Absent		Symptoms Present	
		(*n*)	(%)	(*n*)	(%)
		36	47%	40	53%
**General Characteristics of Concurrent COVID-19 and Pregnancy**					
Number of times pregnancy is documented in the patient’s EHR (including current/recent pregnancy, previous pregnancies, live births, miscarriages, stillbirths or abortions)	1	7	19.44%	2	5.00%
	2	11	30.56%	10	25.00%
	3	5	13.89%	8	20.00%
	4	9	25.00%	6	15.00%
	5	3	8.33%	6	15.00%
	>5	1	2.78%	8	20.00%
Number of multiple gestations BEFORE pregnancy during COVID-19		1	3.45%	1	2.50%
Prior Vaginal Birth		16	55.17%	16	40.00%
**Adverse Outcomes of Prior Pregnancies**					
Miscarriages	1	2	6.90%	9	23.68%
	2	1	3.45%	2	5.26%
	3	0	0.00%	3	7.89%
Still Birth		0	0.00%	1	2.63%
HELLP		0	0.00%	1	2.63%
Cesarean Section (CS) Delivery		7	24.14%	15	39.47%
Gestational Diabetes		0	0.00%	4	10.53%
Gestational Hypertension		3	10.34%	6	15.79%
Preeclampsia		5	17.24%	3	7.89%
Preterm birth		2	6.90%	5	13.16%
**COVID-19 Experience**					
Stage at which acute COVID-19 was diagnosed	Pregnant	20	55.56%	31	77.50%
	In labor/at delivery	16	44.44%	9	22.50%
Treated for acute COVID-19		10	27.78%	15	37.50%
Acute COVID-19 treatment given	Pain relievers and symptom relief	0	0.00%	3	20.00%
	Antivirals	2	20.00%	7	46.67%
	Steroids	2	20.00%	4	26.67%
	Antithrombolytics	7	70.00%	6	40.00%
	Antibiotics	2	20.00%	1	6.67%
	Monoclonal Antibody	1	10.00%	3	20.00%
No documented treatment for acute COVID-19		26	72.22%	25	62.50%
Documented reasons for lack of acute COVID-19 treatment	Asymptomatic	8	30.77%	5	20.00%
	Patient declined	1	3.85%	4	16.00%
	Mild Symptoms	4	15.38%	2	8.00%
Patient hospitalized and/or given oxygen due to acute COVID-19		3	8.33%	1	2.25%
**Pregnancy Outcomes**					
Number of multiple gestations DURING pregnancy during COVID-19		3	8.33%	1	2.50%
Live Birth		32	89%	40	100%
Number of babies	1	30	93.75%	39	97.50%
	2	3	9.38%	1	2.50%
Sex distribution of neonate	M	21	64.00%	20	50.00%
	F	12	36.00%	20	50.00%
Miscarriages		0	0.00%	0	0.00%
Still Birth		2	5.56%	0	0.00%
Preterm birth		11	30.56%	13	32.50%
CS Delivery		15	46.88%	23	57.50%
Reason for CS Delivery	Planned cesarean delivery because of prior cesarean delivery	6	40.00%	9	39.13%
	Abnormal progress in labor	4	26.67%	4	17.39%
	Concern about baby based on the heart monitor	2	13.33%	7	30.43%
	Baby was breech	2	13.33%	2	8.70%
	Emergency due to risk to baby or participant	3	20.00%	3	13.04%
	Patient too sick with COVID-19 to be in labor	1	6.67%	0	0.00%
	Other reasons for a Cesarean	0	0.00%	3	13.04%
Forceps or Vacuum-assisted delivery		0	0.00%	6	15.00%
Gestational diabetes		6	16.67%	3	7.50%
Gestational hypertension		2	5.56%	7	17.50%
Preeclampsia		8	22.22%	8	20.00%
HELLP syndrome		1	2.78%	2	5.00%
Seizures		1	2.78%	0	0.00%
Placenta abruption		3	8.33%	1	2.50%
Preterm premature rupture of membranes		6	16.67%	5	12.50%
Oligohydramnios		1	2.78%	1	2.50%
Hemorrhage or excessive bleeding		4	11.76%	3	7.50%
Blood transfusion		1	2.94%	3	7.50%
Uterine infection		4	11.76%	3	7.50%
Blood clot in the legs of lungs requiring treatment with blood thinning medications		0	0.00%	1	2.50%
Mechanical Ventilation		1	2.94%	0	0.00%
Pneumonia		1	2.94%	2	5.00%
Fetal Growth Restriction		0	0.00%	0	0.00%
Congenital Anomalies		1	3%	1	3%
	Before COVID-19 Diagnosis	1	100%	0	0%
	After COVID-19 Diagnosis	0	0%	1	100%
Category of Congenital Anomaly	Abdomen	0	0%	1	100%
	Brain	0	0%	0	0%
	Face or lip	0	0%	0	0%
	Cardiac	1	100%	0	0%
Trimester anomaly was observed	2nd	1	100%	0	0%
	3rd	0	0%	0	0%
	At birth	0	0%	1	100%
Number of multiple gestations AFTER pregnancy during COVID-19		0	0.00%	0	0.00%
Number of participants currently pregnant		1	2.78%	6	15.00%
**Neonatal Outcomes**					
Neonatal Intensive Care Unit (NICU) admittance		11	34.38%	13	32.50%
Non-Survival at discharge		6	18.75%	1	2.50%
Non-Survival post-partum		1	3.85%	3	7.69%
Average Birth Weight (for those born between 37 and 41 weeks of gestation)		6.0 lbs 7.6 oz		5.8 lbs 6.2 oz	
Average Birth Weight (for all live births)		6 lbs 7.6 oz		5.8 lbs 6.0 oz	

**Table 4:** Characteristics and Outcomes Associated with Pregnancy Based on PASC Score Category.

		PASC 2023 = 0		PASC Indeterminate 2023		PASC 2024 = 0		PASC Indeterminate 2024	
		(*n*)	(%)	(*n*)	(%)	(*n*)	(%)	(*n*)	(%)
		133	89%	15	10%	126	85%	22	15%
**General Characteristics of Concurrent COVID-19 and Pregnancy**									
Number of times pregnancy is documented in the patient’s EHR (including current/recent pregnancy, previous pregnancies, live births, miscarriages, stillbirths or abortions)	1	17	12.78%	0	0.00%	15	11.90%	2	9.090%
	2	33	24.81%	4	26.67%	30	23.81%	7	31.82%
	3	22	16.54%	3	20%	19	15.08%	6	27.27%
	4	27	20.30%	2	13.33%	27	21.43%	3	13.64%
	5	13	9.77%	3	20%	13	10.32%	3	13.64%
	>5	21	15.79%	3	20%	23	18.25%	1	4.55%
Number of multiple gestations BEFORE pregnancy during COVID-19		1	0.86%	1	6.67%	1	0.89%	1	5.00%
Prior Vaginal Birth		57	49.14%	5	33.33%	57	50.89%	5	25.00%
**Adverse Outcomes of Prior Pregnancies**									
Miscarriages	1	29	25.00%	2	13.33%	29	25.89%	2	10.00%
	2	5	4.31%	1	6.67%	5	4.46%	1	5.00%
	3	6	5.17%	1	6.67%	7	6.25%	0	0.00%
Still Birth		1	0.86%	1	6.67%	5	4.46%	1	5.00%
HELLP		0	0.00%	1	6.67%	0	0.00%	1	5.00%
Cesarean Section (CS) Delivery		41	35.34%	5	33.33%	38	33.93%	8	40.00%
Gestational Diabetes		5	4.31%	3	20.00%	5	4.46%	3	15.00%
Gestational Hypertension		12	10.34%	3	20.00%	11	9.82%	4	20.00%
Preeclampsia		16	13.79%	2	13.33%	16	14.29%	2	10.00%
Fetal Growth Restriction		3	2.59%	0	0.00%	3	2.68%	0	0.00%
Preterm birth		13	11.21%	3	20.00%	37	33.04%	2	10.00%
**COVID-19 Experience**									
Stage at which acute COVID-19 was diagnosed	Pregnant	89	66.92%	12	80%	82	65.08%	19	86.36%
	In labor/at delivery	44	33.08%	3	20%	44	34.92%	3	13.64%
Treated for acute COVID-19		41	30.83%	6	40%	40	31.75	6	27.27
Acute COVID-19 treatment given	Pain relievers and symptom relief	6	14.63%	1	16.67%	7	17.50%	0	0.00%
	Antivirals	13	31.71%	3	50.00%	8	20.00%	4	66.67%
	Steroids	7	17.07%	2	33.33%	7	17.5%	2	33.33%
	Antithrombolytics	26	63.41%	2	33.33%	22	55.00%	2	33.33%
	Antibiotics	3	7.32%	0	0.00%	3	7.50%	0	0.00%
	Monoclonal Antibody	7	17.07%	1	16.67%	7	17.5%	1	16.67%
Not documented treatment for acute COVID-19		92	69.17%	9	60.00%	81	64.29%	16	72.73%
Documented reasons for lack of acute COVID-19 treatment	Asymptomatic	30	32.61%	1	11.11%	25	30.86%	4	25.00%
	Patient declined	7	7.61%	3	33.33%	6	7.41%	3	18.75%
	Mild Symptoms	10	10.87%	0	0.00%	9	11.11%	1	6.25%
Patient hospitalized and/or given oxygen due to acute COVID-19		4	3.00%	1	6.67%	4	3.17%	1	4.55%
**Pregnancy Outcomes**									
Number of multiple gestations DURING pregnancy during COVID-19		6	4.51%	1	6.67%	5	3.97%	2	9.09%
Live Birth		126	95%	15	100.00%	118	94.00%	21	95.45%
Number of babies	1	121	96.03%	14	93.33%	114	96.61%	20	95.24%
	2	5	3.97%	1	6.67%	5	4.24%	1	4.76%
Sex distribution of neonate	M	64	51.00%	10	66.67%	61	50.41%	13	59.09%
	F	62	49.00%	5	33.33%	60	49.59%	9	40.91%
Miscarriage		0	0.00%	0	0.00%	0	0.00%	0	0.00%
Still Birth		3	2.26%	0	0.00%	3	2.38%	0	0.00%
Preterm birth		39	29.32%	2	13.33%	37	29.37%	4	8.18%
CS Delivery		58	46.03%	10	66.67%	55	46.61%	13	61.90%
Reason for CS Delivery	Planned cesarean delivery because of prior cesarean delivery	25	43.10%	4	40.00%	22	40.00%	7	53.85%
	Abnormal progress in labor	13	22.41%	1	10.00%	13	23.64%	1	7.69%
	Concern about baby based on the heart monitor	12	20.69%	3	30.00%	12	21.82%	3	23.08%
	Baby was breech	3	5.17%	1	10.00%	3	5.45%	1	7.69%
	Uterine infection	10	17.24%	2	20.00%	7	12.73%	3	23.08%
	Emergency due to risk to baby or participant	10	17.24%	0	0.00%	10	18.18%	0	0.00%
	Patient too sick with COVID-19 to be in labor	1	1.72%	0	0.00%	1	1.82%	0	0.00%
	Other reason(s)	6	10.34%	0	0.00%	6	10.91%	0	0.00%
Forceps or Vacuum-assisted delivery		9	7.14%	2	13.33%	7	5.93%	4	19.05%
Gestational diabetes		15	11.28%	2	13.33%	15	11.90%	2	9.09%
Gestational hypertension		15	11.28%	2	13.33%	15	11.90%	2	9.09%
Preeclampsia		29	21.80%	1	6.67%	27	21.43%	3	13.64%
HELLP syndrome		2	1.50%	1	6.67%	2	1.59%	1	4.55%
Seizures		1	0.75%	0	0.00%	1	0.79%	0	0.00%
Placenta abruption		5	3.76%	0	0.00%	5	3.97%	0	0.00%
Preterm premature rupture of membranes		14	10.53%	1	6.67%	13	10.32%	2	9.09%
Oligohydramnios		2	1.50%	1	6.67%	3	2.38%	0	0.00%
Hemorrhage or excessive bleeding		12	9.30%	1	6.67%	11	9.17%	2	9.52%
Blood transfusion		4	3.10%	1	6.67%	2	1.67%	3	14.29%
Blood clot in the legs of lungs requiring treatment with blood thinning medications		1	0.78%	0	0.00%	1	0.83%	0	0.00%
Mechanical Ventilation		1	0.78%	0	0.00%	1	0.83%	1	4.76%
Pneumonia		2	1.55%	1	6.67%	1	0.83%	2	9.52%
Fetal Growth Restriction		0	0.00%	0	0.00%	0	0.00%	0	0.00%
Congenital Anomalies		8	6.02%	0	0.00%	7	5.56%	1	4.55%
Before COVID-19 diagnosis		7	87.50%	0	0.00%	6	85.71%	1	100%
After COVID-19 Diagnosis		1	12.50%	0	0.00%	1	14.29%	0	0%
Category of Congenital Anomaly	Abdomen	1	12.50%	0	0.00%	1	14.29%	0	0%
	Brain	1	12.50%	0	0.00%	1	14.29%	0	0%
	Face or lip	1	12.50%	0	0.00%	1	14.29%	0	0%
	Cardiac	5	62.50%	0	0.00%	3	42.86%	1	100%
	Lungs	1	12.50%	0	0.00%	1	14.29%	0	0%
	Limbs	1	12.50%	0	0.00%	1	14.29%	0	0%
Trimester anomaly was observed	2nd	5	62.50%	0	0.00%	4	57.14%	1	100%
	3rd	0	0%	0	0.00%	0	0%	0	0%
	At birth	1	12.50%	0	0.00%	1	14.29%	0	0%
Number of multiple gestations AFTER pregnancy during COVID-19		1	0.75%	0	0.00%	1	0.79%	0	0.00%
Number of participants currently pregnant		5	3.76%	4	26.67%	6	4.76%	3	13.64%
**Neonatal Outcomes**									
Neonatal Intensive Care Unit (NICU) admittance		37	29.37%	5	33.33%	36	30.51%	6	28.57%
Non-Survival at discharge		12	9.52%	0	0.00%	11	9.32%	1	4.76%
Non-Survival post-partum		4	3.51%	2	13.33%	4	3.74%	2	9.52%
Average Birth Weight (for those born between 37 and 41 weeks of gestation)		5.9 lb 8.2 oz		6.2 lbs 5.4 oz		5.8 lbs 8.2 oz		5.8 lbs 8.2 oz	
Average Birth Weight (for all live births)		5.8 lbs 8.9 oz		5.9 lbs 6.1 oz		5.9 lbs 7.6 oz		5.7 lbs 7.3 oz	

## Data Availability

The raw data supporting the conclusions of this article will be made available by the authors on request.

## Supplementary Materials

The following supporting information can be downloaded at: https://www.mdpi.com/article/10.3390/jcm14217869/s1, Table S1: Details of group assignment based on symptom documentation over time. Figure S1: Long COVID Data Collection Instruments.
