# Supplementary material for: A Cohort Study Characterizing the Outcomes Following an Acute SARS-CoV-2 Infection in Pregnancy
Source: J Clin Med. 2025 Nov 6;14(21):7869. doi: 10.3390/jcm14217869 (PMC12610175; doi:10.3390/jcm14217869)
Supplement: Supplementary file 1 [file jcm-14-07869-s001.zip › jcm-3930430-Supplemental Material.pdf]

Pasc Symptoms_EHR v.5

Record ID ______________________________

In the EHR document, in general, how would you rate your physical health? (as of last reviewed encounter)
- ☐ Good (characterized by a check up visit reporting such)
- ☐ Fair (characterized by an acute event, outside of which the patient is well)
- ☐ Poor (characterized by 2 or more chronic events)

What date is this encounter? ______________________________

What kind of encounter ______________________________

In the EHR documentation, in general, how would you rate your mental health, including your mood and your ability to think?
- ☐ Good (a check up encounter where a patient is reported as well)
- ☐ Fair (an acute event or traumatic event has negatively altered a patient's mental state)
- ☐ Poor (chronic mental issues)

What date is this encounter? ______________________________

What kind of encounter ______________________________

Poor appetite or overeating:
- ☐ Yes, it is documented in EHR in the YEAR BEFORE COVID
- ☐ Yes, it is documented BETWEEN 30 DAYS AFTER COVID but no longer with most recent encounter
- ☐ Yes, it is documented in EHR BETWEEN 30 DAYS AFTER COVID AND NOW
- ☐ Yes, it is documented in EHR NOW
- ☐ Yes, I DID have it BETWEEN 22 DAYS AFTER COVID but no longer have it
- ☐ Yes, it is documented in EHR BETWEEN 22 DAYS AFTER COVID AND NOW
- ☐ No, this is not documented as occurring in any the above windows of time

"What is the date of the initial onset during the "year before" period?" ______________________________

What kind of encounter ______________________________

What is the date of initial onset of post-acute COVID? ______________________________

What kind of encounter ______________________________

Fatigue (being very tired)	☐ Yes, it is documented in EHR in the YEAR BEFORE COVID<br>☐ Yes, it is documented BETWEEN 30 DAYS AFTER COVID but no longer with most recent encounter<br>☐ Yes, it is documented in EHR BETWEEN 30 DAYS AFTER COVID AND NOW<br>☐ Yes, it is documented in EHR NOW<br>☐ Yes, I DID have it BETWEEN 22 DAYS AFTER COVID but no longer have it<br>☐ Yes, it is documented in EHR BETWEEN 22 DAYS AFTER COVID AND NOW<br>☐ No, this is not documented as occurring in any the above windows of time
"What is the date of the initial onset during the "year before" period?"	________________________
What kind of encounter	________________________
What is the date of initial onset of post-acute COVID?	________________________
What kind of encounter	________________________
Post-exertional malaise (Symptoms worse after even minor physical or mental effort)	☐ Yes, it is documented in EHR in the YEAR BEFORE COVID<br>☐ Yes, it is documented BETWEEN 30 DAYS AFTER COVID but no longer with most recent encounter<br>☐ Yes, it is documented in EHR BETWEEN 30 DAYS AFTER COVID AND NOW<br>☐ Yes, it is documented in EHR NOW<br>☐ Yes, I DID have it BETWEEN 22 DAYS AFTER COVID but no longer have it<br>☐ Yes, it is documented in EHR BETWEEN 22 DAYS AFTER COVID AND NOW<br>☐ No, this is not documented as occurring in any the above windows of time
"What is the date of the initial onset during the "year before" period?"	________________________
What kind of encounter	________________________
What is the date of initial onset of post-acute COVID?	________________________
What kind of encounter	________________________

Swelling of your legs, Weakness in arms or legs, muscle cramps in your legs and/or feet?	☐ Yes, it is documented in EHR in the YEAR BEFORE COVID<br>☐ Yes, it is documented BETWEEN 30 DAYS AFTER COVID but no longer with most recent encounter<br>☐ Yes, it is documented in EHR BETWEEN 30 DAYS AFTER COVID AND NOW<br>☐ Yes, it is documented in EHR NOW<br>☐ Yes, I DID have it BETWEEN 22 DAYS AFTER COVID but no longer have it<br>☐ Yes, it is documented in EHR BETWEEN 22 DAYS AFTER COVID AND NOW<br>☐ No, this is not documented as occurring in any the above windows of time
"What is the date of the initial onset during the "year before" period?"	______________________
What kind of encounter	______________________
What is the date of initial onset of post-acute COVID?	______________________
What kind of encounter	______________________
Fever, chills, sweats or flushing	☐ Yes, it is documented in EHR in the YEAR BEFORE COVID<br>☐ Yes, it is documented BETWEEN 30 DAYS AFTER COVID but no longer with most recent encounter<br>☐ Yes, it is documented in EHR BETWEEN 30 DAYS AFTER COVID AND NOW<br>☐ Yes, it is documented in EHR NOW<br>☐ Yes, I DID have it BETWEEN 22 DAYS AFTER COVID but no longer have it<br>☐ Yes, it is documented in EHR BETWEEN 22 DAYS AFTER COVID AND NOW<br>☐ No, this is not documented as occurring in any the above windows of time
"What is the date of the initial onset during the "year before" period?"	______________________
What kind of encounter	______________________
What is the date of initial onset of post-acute COVID?	______________________
What kind of encounter	______________________

Loss of or change in smell or taste	☐ Yes, it is documented in EHR in the YEAR BEFORE COVID<br>☐ Yes, it is documented BETWEEN 30 DAYS AFTER COVID but no longer with most recent encounter<br>☐ Yes, it is documented in EHR BETWEEN 30 DAYS AFTER COVID AND NOW<br>☐ Yes, it is documented in EHR NOW<br>☐ Yes, I DID have it BETWEEN 22 DAYS AFTER COVID but no longer have it<br>☐ Yes, it is documented in EHR BETWEEN 22 DAYS AFTER COVID AND NOW<br>☐ No, this is not documented as occurring in any the above windows of time
"What is the date of the initial onset during the "year before" period?"	________________________________
What kind of encounter	________________________________
What is the date of initial onset of post-acute COVID?	________________________________
What kind of encounter	________________________________
Pain in any part of your body	☐ Yes, it is documented in EHR in the YEAR BEFORE COVID<br>☐ Yes, it is documented BETWEEN 30 DAYS AFTER COVID but no longer with most recent encounter<br>☐ Yes, it is documented in EHR BETWEEN 30 DAYS AFTER COVID AND NOW<br>☐ Yes, it is documented in EHR NOW<br>☐ Yes, I DID have it BETWEEN 22 DAYS AFTER COVID but no longer have it<br>☐ Yes, it is documented in EHR BETWEEN 22 DAYS AFTER COVID AND NOW<br>☐ No, this is not documented as occurring in any the above windows of time
Check all that apply	☐ Head pain/headache<br>☐ Chest pain (including chest tightness, pressure)<br>☐ Abdomen (belly)<br>☐ Pelvis or genitals<br>☐ Joints<br>☐ Muscles<br>☐ Back/spine<br>☐ Skin<br>☐ Feet<br>☐ Mouth<br>☐ Throat
"What is the date of the initial onset during the "year before" period?"	________________________________
What kind of encounter	________________________________
What is the date of initial onset of post-acute COVID?	________________________________

What kind of encounter

____

Shortness of breath

- ☐ Yes, it is documented in EHR in the YEAR BEFORE COVID
- ☐ Yes, it is documented BETWEEN 30 DAYS AFTER COVID but no longer with most recent encounter
- ☐ Yes, it is documented in EHR BETWEEN 30 DAYS AFTER COVID AND NOW
- ☐ Yes, it is documented in EHR NOW
- ☐ Yes, I DID have it BETWEEN 22 DAYS AFTER COVID but no longer have it
- ☐ Yes, it is documented in EHR BETWEEN 22 DAYS AFTER COVID AND NOW
- ☐ No, this is not documented as occurring in any the above windows of time

"What is the date of the initial onset during the "year before" period?"

____

What kind of encounter

____

What is the date of initial onset of post-acute COVID?

____

What kind of encounter

____

Cough

- ☐ Yes, it is documented in EHR in the YEAR BEFORE COVID
- ☐ Yes, it is documented BETWEEN 30 DAYS AFTER COVID but no longer with most recent encounter
- ☐ Yes, it is documented in EHR BETWEEN 30 DAYS AFTER COVID AND NOW
- ☐ Yes, it is documented in EHR NOW
- ☐ Yes, I DID have it BETWEEN 22 DAYS AFTER COVID but no longer have it
- ☐ Yes, it is documented in EHR BETWEEN 22 DAYS AFTER COVID AND NOW
- ☐ No, this is not documented as occurring in any the above windows of time

"What is the date of the initial onset during the "year before" period?"

____

What kind of encounter

____

What is the date of initial onset of post-acute COVID?

____

What kind of encounter

____

Palpitations, racing heart, arrhythmia, skipped beats	☐ Yes, it is documented in EHR in the YEAR BEFORE COVID<br>☐ Yes, it is documented BETWEEN 30 DAYS AFTER COVID but no longer with most recent encounter<br>☐ Yes, it is documented in EHR BETWEEN 30 DAYS AFTER COVID AND NOW<br>☐ Yes, it is documented in EHR NOW<br>☐ Yes, I DID have it BETWEEN 22 DAYS AFTER COVID but no longer have it<br>☐ Yes, it is documented in EHR BETWEEN 22 DAYS AFTER COVID AND NOW<br>☐ No, this is not documented as occurring in any the above windows of time
"What is the date of the initial onset during the "year before" period?"	______________________
What kind of encounter	______________________
What is the date of initial onset of post-acute COVID?	______________________
What kind of encounter	______________________
Gastrointestinal (belly) symptoms (feeling full or vomiting after eating, diarrhea, constipation, cramping or colicky abdominal pain)?	☐ Yes, it is documented in EHR in the YEAR BEFORE COVID<br>☐ Yes, it is documented BETWEEN 30 DAYS AFTER COVID but no longer with most recent encounter<br>☐ Yes, it is documented in EHR BETWEEN 30 DAYS AFTER COVID AND NOW<br>☐ Yes, it is documented in EHR NOW<br>☐ Yes, I DID have it BETWEEN 22 DAYS AFTER COVID but no longer have it<br>☐ Yes, it is documented in EHR BETWEEN 22 DAYS AFTER COVID AND NOW<br>☐ No, this is not documented as occurring in any the above windows of time
"What is the date of the initial onset during the "year before" period?"	______________________
What kind of encounter	______________________
What is the date of initial onset of post-acute COVID?	______________________
What kind of encounter	______________________

Bladder problems (incontinence, trouble passing urine or emptying bladder)	☐ Yes, it is documented in EHR in the YEAR BEFORE COVID<br>☐ Yes, it is documented BETWEEN 30 DAYS AFTER COVID but no longer with most recent encounter<br>☐ Yes, it is documented in EHR BETWEEN 30 DAYS AFTER COVID AND NOW<br>☐ Yes, it is documented in EHR NOW<br>☐ Yes, I DID have it BETWEEN 22 DAYS AFTER COVID but no longer have it<br>☐ Yes, it is documented in EHR BETWEEN 22 DAYS AFTER COVID AND NOW<br>☐ No, this is not documented as occurring in any the above windows of time
"What is the date of the initial onset during the "year before" period?"	______________________________
What kind of encounter	______________________________
What is the date of initial onset of post-acute COVID?	______________________________
What kind of encounter	______________________________
Nerve problems (tremor, shaking, abnormal movements, numbness, tingling, burning, can't move part of body, new seizures)	☐ Yes, it is documented in EHR in the YEAR BEFORE COVID<br>☐ Yes, it is documented BETWEEN 30 DAYS AFTER COVID but no longer with most recent encounter<br>☐ Yes, it is documented in EHR BETWEEN 30 DAYS AFTER COVID AND NOW<br>☐ Yes, it is documented in EHR NOW<br>☐ Yes, I DID have it BETWEEN 22 DAYS AFTER COVID but no longer have it<br>☐ Yes, it is documented in EHR BETWEEN 22 DAYS AFTER COVID AND NOW<br>☐ No, this is not documented as occurring in any the above windows of time
"What is the date of the initial onset during the "year before" period?"	______________________________
What kind of encounter	______________________________
What is the date of initial onset of post-acute COVID?	______________________________
What kind of encounter	______________________________

Problems with anxiety, depression, stress, or trauma-related symptoms like nightmares or grief	☐ Yes, it is documented in EHR in the YEAR BEFORE COVID<br>☐ Yes, it is documented BETWEEN 30 DAYS AFTER COVID but no longer with most recent encounter<br>☐ Yes, it is documented in EHR BETWEEN 30 DAYS AFTER COVID AND NOW<br>☐ Yes, it is documented in EHR NOW<br>☐ Yes, I DID have it BETWEEN 22 DAYS AFTER COVID but no longer have it<br>☐ Yes, it is documented in EHR BETWEEN 22 DAYS AFTER COVID AND NOW<br>☐ No, this is not documented as occurring in any the above windows of time
"What is the date of the initial onset during the "year before" period?"	______________________
What kind of encounter	______________________
What is the date of initial onset of post-acute COVID?	______________________
What kind of encounter	______________________
Problems with sleep	☐ Yes, it is documented in EHR in the YEAR BEFORE COVID<br>☐ Yes, it is documented BETWEEN 30 DAYS AFTER COVID but no longer with most recent encounter<br>☐ Yes, it is documented in EHR BETWEEN 30 DAYS AFTER COVID AND NOW<br>☐ Yes, it is documented in EHR NOW<br>☐ Yes, I DID have it BETWEEN 22 DAYS AFTER COVID but no longer have it<br>☐ Yes, it is documented in EHR BETWEEN 22 DAYS AFTER COVID AND NOW<br>☐ No, this is not documented as occurring in any the above windows of time
"What is the date of the initial onset during the "year before" period?"	______________________
What kind of encounter	______________________
What is the date of initial onset of post-acute COVID?	______________________
What kind of encounter	______________________

Problems thinking or concentrating ("brain fog"), Feeling faint, dizzy, "goofy"; difficulty thinking soon after standing up from a sitting or lying position	☐ Yes, it is documented in EHR in the YEAR BEFORE COVID<br>☐ Yes, it is documented BETWEEN 30 DAYS AFTER COVID but no longer with most recent encounter<br>☐ Yes, it is documented in EHR BETWEEN 30 DAYS AFTER COVID AND NOW<br>☐ Yes, it is documented in EHR NOW<br>☐ Yes, I DID have it BETWEEN 22 DAYS AFTER COVID but no longer have it<br>☐ Yes, it is documented in EHR BETWEEN 22 DAYS AFTER COVID AND NOW<br>☐ No, this is not documented as occurring in any the above windows of time
"What is the date of the initial onset during the "year before" period?"	__________________________________
What kind of encounter	__________________________________
What is the date of initial onset of post-acute COVID?	__________________________________
What kind of encounter	__________________________________
Color changes in your skin, such as red, white or purple	☐ Yes, it is documented in EHR in the YEAR BEFORE COVID<br>☐ Yes, it is documented BETWEEN 30 DAYS AFTER COVID but no longer with most recent encounter<br>☐ Yes, it is documented in EHR BETWEEN 30 DAYS AFTER COVID AND NOW<br>☐ Yes, it is documented in EHR NOW<br>☐ Yes, I DID have it BETWEEN 22 DAYS AFTER COVID but no longer have it<br>☐ Yes, it is documented in EHR BETWEEN 22 DAYS AFTER COVID AND NOW<br>☐ No, this is not documented as occurring in any the above windows of time
"What is the date of the initial onset during the "year before" period?"	__________________________________
What kind of encounter	__________________________________
What is the date of initial onset of post-acute COVID?	__________________________________
What kind of encounter	__________________________________
In the EHR documentation, what parts of the patient's body are affected by these color changes? (check all that apply)	☐ Hands<br>☐ Feet<br>☐ Other<br>☐ No, this is not documented

Skin rash, sores	☐ Yes, it is documented in EHR in the YEAR BEFORE COVID<br>☐ Yes, it is documented BETWEEN 30 DAYS AFTER COVID but no longer with most recent encounter<br>☐ Yes, it is documented in EHR BETWEEN 30 DAYS AFTER COVID AND NOW<br>☐ Yes, it is documented in EHR NOW<br>☐ Yes, I DID have it BETWEEN 22 DAYS AFTER COVID but no longer have it<br>☐ Yes, it is documented in EHR BETWEEN 22 DAYS AFTER COVID AND NOW<br>☐ No, this is not documented as occurring in any the above windows of time
"What is the date of the initial onset during the "year before" period?"	__________________________________
What kind of encounter	__________________________________
What is the date of initial onset of post-acute COVID?	__________________________________
What kind of encounter	__________________________________
Excessively dry mouth, eyes?	☐ Yes, it is documented in EHR in the YEAR BEFORE COVID<br>☐ Yes, it is documented BETWEEN 30 DAYS AFTER COVID but no longer with most recent encounter<br>☐ Yes, it is documented in EHR BETWEEN 30 DAYS AFTER COVID AND NOW<br>☐ Yes, it is documented in EHR NOW<br>☐ Yes, I DID have it BETWEEN 22 DAYS AFTER COVID but no longer have it<br>☐ Yes, it is documented in EHR BETWEEN 22 DAYS AFTER COVID AND NOW<br>☐ No, this is not documented as occurring in any the above windows of time
"What is the date of the initial onset during the "year before" period?"	__________________________________
What kind of encounter	__________________________________
What is the date of initial onset of post-acute COVID?	__________________________________
What kind of encounter	__________________________________

Excessive thirst	☐ Yes, it is documented in EHR in the YEAR BEFORE COVID<br>☐ Yes, it is documented BETWEEN 30 DAYS AFTER COVID but no longer with most recent encounter<br>☐ Yes, it is documented in EHR BETWEEN 30 DAYS AFTER COVID AND NOW<br>☐ Yes, it is documented in EHR NOW<br>☐ Yes, I DID have it BETWEEN 22 DAYS AFTER COVID but no longer have it<br>☐ Yes, it is documented in EHR BETWEEN 22 DAYS AFTER COVID AND NOW<br>☐ No, this is not documented as occurring in any the above windows of time
"What is the date of the initial onset during the "year before" period?"	______________________
What kind of encounter	______________________
What is the date of initial onset of post-acute COVID?	______________________
What kind of encounter	______________________
Vision problems (blurry, light sensitivity, difficulty reading or focusing, floaters, flashing lights, "snow")	☐ Yes, it is documented in EHR in the YEAR BEFORE COVID<br>☐ Yes, it is documented BETWEEN 30 DAYS AFTER COVID but no longer with most recent encounter<br>☐ Yes, it is documented in EHR BETWEEN 30 DAYS AFTER COVID AND NOW<br>☐ Yes, it is documented in EHR NOW<br>☐ Yes, I DID have it BETWEEN 22 DAYS AFTER COVID but no longer have it<br>☐ Yes, it is documented in EHR BETWEEN 22 DAYS AFTER COVID AND NOW<br>☐ No, this is not documented as occurring in any the above windows of time
"What is the date of the initial onset during the "year before" period?"	______________________
What kind of encounter	______________________
What is the date of initial onset of post-acute COVID?	______________________
What kind of encounter	______________________

Problems with hearing (hearing loss, ringing in ears)	☐ Yes, it is documented in EHR in the YEAR BEFORE COVID<br>☐ Yes, it is documented BETWEEN 30 DAYS AFTER COVID but no longer with most recent encounter<br>☐ Yes, it is documented in EHR BETWEEN 30 DAYS AFTER COVID AND NOW<br>☐ Yes, it is documented in EHR NOW<br>☐ Yes, I DID have it BETWEEN 22 DAYS AFTER COVID but no longer have it<br>☐ Yes, it is documented in EHR BETWEEN 22 DAYS AFTER COVID AND NOW<br>☐ No, this is not documented as occurring in any the above windows of time
"What is the date of the initial onset during the "year before" period?"	__________________________
What kind of encounter	__________________________
What is the date of initial onset of post-acute COVID?	__________________________
What kind of encounter	__________________________
Hair loss	☐ Yes, it is documented in EHR in the YEAR BEFORE COVID<br>☐ Yes, it is documented BETWEEN 30 DAYS AFTER COVID but no longer with most recent encounter<br>☐ Yes, it is documented in EHR BETWEEN 30 DAYS AFTER COVID AND NOW<br>☐ Yes, it is documented in EHR NOW<br>☐ Yes, I DID have it BETWEEN 22 DAYS AFTER COVID but no longer have it<br>☐ Yes, it is documented in EHR BETWEEN 22 DAYS AFTER COVID AND NOW<br>☐ No, this is not documented as occurring in any the above windows of time
"What is the date of the initial onset during the "year before" period?"	__________________________
What kind of encounter	__________________________
What is the date of initial onset of post-acute COVID?	__________________________
What kind of encounter	__________________________

Problems with teeth

- ☐ Yes, it is documented in EHR in the YEAR BEFORE COVID
- ☐ Yes, it is documented BETWEEN 30 DAYS AFTER COVID but no longer with most recent encounter
- ☐ Yes, it is documented in EHR BETWEEN 30 DAYS AFTER COVID AND NOW
- ☐ Yes, it is documented in EHR NOW
- ☐ Yes, I DID have it BETWEEN 22 DAYS AFTER COVID but no longer have it
- ☐ Yes, it is documented in EHR BETWEEN 22 DAYS AFTER COVID AND NOW
- ☐ No, this is not documented as occurring in any the above windows of time

"What is the date of the initial onset during the "year before" period?" ______________________________

What kind of encounter ______________________________

What is the date of initial onset of post-acute COVID? ______________________________

What kind of encounter ______________________________

Changes to menstrual cycle, reports of heavy periods

- ☐ Yes, it is documented in EHR in the YEAR BEFORE COVID
- ☐ Yes, it is documented BETWEEN 30 DAYS AFTER COVID but no longer with most recent encounter
- ☐ Yes, it is documented in EHR BETWEEN 30 DAYS AFTER COVID AND NOW
- ☐ Yes, it is documented in EHR NOW
- ☐ Yes, I DID have it BETWEEN 22 DAYS AFTER COVID but no longer have it
- ☐ Yes, it is documented in EHR BETWEEN 22 DAYS AFTER COVID AND NOW
- ☐ No, this is not documented as occurring in any the above windows of time

"What is the date of the initial onset during the "year before" period?" ______________________________

What kind of encounter ______________________________

What is the date of initial onset of post-acute COVID? ______________________________

What kind of encounter ______________________________

Changes in fertility or difficulty getting pregnant	☐ Yes, it is documented in EHR in the YEAR BEFORE COVID<br>☐ Yes, it is documented BETWEEN 30 DAYS AFTER COVID but no longer with most recent encounter<br>☐ Yes, it is documented in EHR BETWEEN 30 DAYS AFTER COVID AND NOW<br>☐ Yes, it is documented in EHR NOW<br>☐ Yes, I DID have it BETWEEN 22 DAYS AFTER COVID but no longer have it<br>☐ Yes, it is documented in EHR BETWEEN 22 DAYS AFTER COVID AND NOW<br>☐ No, this is not documented as occurring in any the above windows of time
"What is the date of the initial onset during the "year before" period?"	________________________
What kind of encounter	________________________
What is the date of initial onset of post-acute COVID?	________________________
What kind of encounter	________________________
In the EHR documentation, are there any other symptoms that are attributed to COVID?	◯ Yes, it is documented in EHR<br>◯ No, it is not documented in EHR
Please specify any other symptoms documented as attributed to COVID:	______________________________
In the EHR, is there documentation that patient has ever had diabetic neuropathy?	☐ Yes, it is documented in EHR in the YEAR BEFORE COVID<br>☐ Yes, it is documented BETWEEN 30 DAYS AFTER COVID but no longer with most recent encounter<br>☐ Yes, it is documented in EHR BETWEEN 30 DAYS AFTER COVID AND NOW<br>☐ Yes, it is documented in EHR NOW<br>☐ Yes, I DID have it BETWEEN 22 DAYS AFTER COVID but no longer have it<br>☐ Yes, it is documented in EHR BETWEEN 22 DAYS AFTER COVID AND NOW<br>☐ No, this is not documented as occurring in any the above windows of time
"What is the date of the initial onset during the "year before" period?"	________________________
What kind of encounter	________________________
What is the date of initial onset of post-acute COVID?	________________________
What kind of encounter	________________________

Are symptoms documented In the EHR, as ever worse at night?	◯ Yes, it is documented<br>◯ No, it is not documented
In the EHR documentation, which symptom type is documented as getting worse at night?	☐ 1. Mental<br>☐ 2. Pain<br>☐ 3. Fatigue<br>☐ 4. Neurological<br>☐ 5. Pulmonary<br>☐ 6. Lymphatic/Inflammatory<br>☐ 7. Gastrointestinal<br>☐ 8. Urinary<br>☐ 9. Cognitive<br>☐ 10. Sleep<br>☐ 11. Dermatological<br>☐ 12. Gustatory<br>☐ 13. Vision<br>☐ 14. Dental<br>☐ 15. Gynecological (Menstrual/Menopause)<br>☐ 16. Fertility
In the EHR documentation, how many symptoms is reported as worse at night?	◯ 1. 1<br>◯ 2. 2<br>◯ 3. 3 or more
In the EHR, is it documented that eyesight using both eyes (with glasses or contact lenses, if worn) is) is good, poor or is there complete blindness?	☐ Good<br>☐ Poor<br>☐ Complete blindness<br>☐ Yes, it is documented in EHR in the YEAR BEFORE COVID<br>☐ Yes, it is documented BETWEEN 30 DAYS AFTER COVID but no longer with most recent encounter<br>☐ Yes, it is documented in EHR BETWEEN 30 DAYS AFTER COVID AND NOW<br>☐ Yes, it is documented in EHR NOW<br>☐ Yes, I DID have it BETWEEN 22 DAYS AFTER COVID but no longer have it<br>☐ Yes, it is documented in EHR BETWEEN 22 DAYS AFTER COVID AND NOW<br>☐ No, this is not documented as occurring in any the above windows of time
"What is the date of the initial onset during the "year before" period?"	______________________________
What kind of encounter	______________________________
What is the date of initial onset of post-acute COVID?	______________________________
What kind of encounter	______________________________

In the EHR documentation, are there reports of hot flashes	☐ Yes, it is documented in EHR in the YEAR BEFORE COVID<br>☐ Yes, it is documented BETWEEN 30 DAYS AFTER COVID but no longer with most recent encounter<br>☐ Yes, it is documented in EHR BETWEEN 30 DAYS AFTER COVID AND NOW<br>☐ Yes, it is documented in EHR NOW<br>☐ Yes, I DID have it BETWEEN 22 DAYS AFTER COVID but no longer have it<br>☐ Yes, it is documented in EHR BETWEEN 22 DAYS AFTER COVID AND NOW<br>☐ No, this is not documented as occurring in any the above windows of time
"What is the date of the initial onset during the "year before" period?"	________________________
What kind of encounter	________________________
What is the date of initial onset of post-acute COVID?	________________________
What kind of encounter	________________________
Is there EHR documentation for any treatment for infertility including medications or procedures such as IVF?	☐ Yes, it is documented in EHR in the YEAR BEFORE COVID<br>☐ Yes, it is documented BETWEEN 30 DAYS AFTER COVID but no longer with most recent encounter<br>☐ Yes, it is documented in EHR BETWEEN 30 DAYS AFTER COVID AND NOW<br>☐ Yes, it is documented in EHR NOW<br>☐ Yes, I DID have it BETWEEN 22 DAYS AFTER COVID but no longer have it<br>☐ Yes, it is documented in EHR BETWEEN 22 DAYS AFTER COVID AND NOW<br>☐ No, this is not documented as occurring in any the above windows of time
"What is the date of the initial onset during the "year before" period?"	________________________
What kind of encounter	________________________
What is the date of initial fertility treatment during the ' between 22 days after and Now'	________________________
What is the date of initial fertility treatment during the ' between 30 days after and Now'	________________________
What kind of encounter	________________________
What is the date of initial fertility treatment NOW?	________________________

What is the end date of the fertility treatment?

____

Is there EHR documentation that the patient has an encounter at the hospital [stem_sincein]? Check all that apply.

- ☐ Yes, an emergency department encounter is documented for labor
- ☐ Yes, a hospital admission is documented is documented for labor
- ☐ Yes, an emergency department encounter is documented for a non-labor incident
- ☐ Yes, a hospital admission is documented is documented for a non-labor incident
- ☐ No, it is not documented

Pregnancy EHR v. 5

Record ID	______________________________
Is pregnancy ever documented in the patient's EHR?	◯ it is documented in EHR<br>◯ it is not documented in EHR
How many times is pregnancy documented in the patient's EHR (including current/recent pregnancy, previous pregnancies, live births, miscarriages, stillbirths or abortions)?	◯ 0<br>◯ 1<br>◯ 2<br>◯ 3<br>◯ 4<br>◯ 5<br>◯ >5<br>◯ not documented
Is there multiple gestations (multiple births at a time) documented in the EHR?	◯ Yes<br>◯ No
If yes, how many pregnancies were multiparous BEFORE pregnancy during COVID	◯ 0<br>◯ 1<br>◯ 2<br>◯ 3<br>◯ 4<br>◯ 5<br>◯ >5<br>◯ not documented
If yes, how many pregnancies were multiparous DURING pregnancy during COVID	◯ 0<br>◯ 1<br>◯ 2<br>◯ 3<br>◯ 4<br>◯ 5<br>◯ >5<br>◯ not documented
If yes, how many pregnancies were multiparous AFTER pregnancy during COVID	◯ 0<br>◯ 1<br>◯ 2<br>◯ 3<br>◯ 4<br>◯ 5<br>◯ >5<br>◯ not documented
In the EHR documentation, how many times has pregnancy resulted in the live birth of a baby?	◯ 0<br>◯ 1<br>◯ 2<br>◯ 3<br>◯ 4<br>◯ 5<br>◯ >5<br>◯ not documented

In the EHR documentation, how many of the pregnancies resulted in a miscarriage?	◯ 0<br>◯ 1<br>◯ 2<br>◯ 3<br>◯ 4<br>◯ 5<br>◯ >5<br>◯ not documented
In the EHR documentation, how many of the pregnancies resulted in a stillbirth (the death of the fetus at more than 20 weeks (5 months) of pregnancy)?	◯ 0<br>◯ 1<br>◯ 2<br>◯ 3<br>◯ 4<br>◯ 5<br>◯ >5<br>◯ not documented
In the EHR documentation, how many of the pregnancies resulted in an abortion?	◯ 0<br>◯ 1<br>◯ 2<br>◯ 3<br>◯ 4<br>◯ 5<br>◯ >5<br>◯ not documented
In the EHR documentation, during any pregnancy BEFORE the COVID diagnosis, were any of these conditions reported:	☐ Diabetes (high blood sugars), pregnancy related (sometimes called gestational diabetes)<br>☐ High blood pressure, pregnancy related (sometimes called gestational hypertension)<br>☐ Preeclampsia (sometimes called "toxemia")<br>☐ HELLP syndrome (abnormal liver function and changes in blood platelet counts, often also with high blood pressure)<br>☐ Preterm birth (baby born more than 3 weeks before the due date)<br>☐ fetal growth restriction<br>☐ None of these conditions<br>☐ not documented<br>☐ No pregnancies BEFORE [stem_my]
In the EHR documentation, is there a record of patient currently pregnant?	◯ Yes, it is documented in EHR<br>◯ No, it is not documented in EHR
What trimester is this documented as?	◯ 1st<br>◯ 2nd<br>◯ 3rd<br>◯ 4th<br>◯ not documented
In the EHR documentation, was the patient pregnant/post partum when they had COVID?	◯ Pregnant<br>◯ Post-partum<br>◯ In labor/At birth<br>◯ It is not documented in EHR

In the EHR documentation, when the patient had COVID during pregnancy, how did the pregnancy end?	☐ Abortion<br>☐ Miscarriage<br>☐ Ectopic pregnancy<br>☐ Molar pregnancy<br>☐ Stillbirth (Death of a fetus >20 weeks (5 months) of pregnancy)<br>☐ Live birth of a baby or babies<br>☐ Not documented in the EHR
In the EHR documentation, how far along in the pregnancy was the patient when the abortion occurred?	◯ 1st trimester<br>◯ 2nd trimester<br>◯ 3rd trimester
In the EHR documentation, how far along in the pregnancy was the patient when the miscarriage occurred?	◯ 1st trimester<br>◯ 2nd trimester<br>◯ not documented
In the EHR documentation, how far along in the pregnancy was the patient when the stillbirth (fetal death) occurred?	◯ 2nd trimester<br>◯ 3rd trimester<br>◯ not documented
In the EHR documentation, when the patient had COVID in pregnancy, what was the due date for the pregnancy?	______________________________
In the EHR documentation, when the patient had COVID in pregnancy, what was the actual date of birth of the<br><br>baby?	______________________________
In the EHR documentation, when the patient had COVID during pregnancy, did they have any of the following conditions (check all that apply):	☐ Diabetes, pregnancy related (gestational diabetes)<br>☐ High blood pressure, pregnancy related (gestational hypertension)<br>☐ Preeclampsia (sometimes called "toxemia")<br>☐ HELLP syndrome (abnormal liver function and low blood platelet levels, often also with high blood pressure)<br>☐ Seizures<br>☐ Placenta abruption (when the placenta separates off from the uterus)<br>☐ Preterm premature rupture of membranes (when the bag of water breaks at a time when the baby would be born premature, eg. before 37 weeks of pregnancy)<br>☐ Low amniotic fluid levels (oligohydramnios)<br>☐ Other (specify)<br>☐ None<br>☐ not documented
Other, please specify:	______________________________
In the EHR documentation, when the patient had COVID during pregnancy, did they receive a steroid shot during pregnancy to get the baby ready for an early delivery (medication called betamethasone or dexamethasone)?	◯ Yes, it is documented in EHR<br>◯ No, it is not documented in EHR
When the patient had COVID during pregnancy, is there documentation of any medication being received?	◯ Yes<br>◯ No

If no, why?	______________________
In the EHR documentation, which treatment/medication did the patient receive?	______________________
In the EHR documentation, how long did the patient take/receive treatment?	______________________
In the EHR documentation, did the COVID illness result in the doctor or midwife delivering the baby before the patient had planned to deliver?	◯ Yes, it is documented in EHR<br>◯ No, it is not documented in EHR
In the EHR documentation, when the patient had COVID in pregnancy, did they have any of the following conditions during or after the birth (check all that apply)	☐ Hemorrhage or excessive bleeding<br>☐ Blood transfusion<br>☐ Uterine infection (also called chorioamnionitis or endometritis) during or after the birth<br>☐ Blood clot in the legs of lungs requiring treatment with blood thinning medications<br>☐ Other (please explain below):<br>☐ pneumonia<br>☐ sepsis<br>☐ mechanical ventilation<br>☐ None<br>☐ not documented
In the EHR documentation, was a prior birth/delivery Caesarian or Vaginal?	◯ Caesarian<br>◯ Vaginal<br>◯ No prior birth/delivery<br>◯ not documented
Other, please specify:	______________________
In the EHR documentation, what is the zipcode for the facility the birth happened?	______________________
In the EHR documentation, how many babies were reported born?	◯ 1<br>◯ 2<br>◯ 3<br>◯ 4<br>◯ >5<br>◯ not documented
In the EHR documentation, was the baby reported born by:	◯ Vaginal delivery<br>◯ Cesarean delivery<br>◯ not documented
In the EHR documentation, was a vacuum (suction cup)or forceps reported as used to deliver the baby?	◯ Yes, it is documented in the EHR<br>◯ No, it is not documented in the EHR

In the EHR documentation, what was the reason the patient had a cesarean delivery?	☐ Planned cesarean delivery because I had a prior cesarean delivery<br>☐ Abnormal progress in labor<br>☐ Concern about your baby based on the heart monitor<br>☐ Baby was breech<br>☐ Uterine infection<br>☐ Emergency due to risk to baby or myself<br>☐ I was too sick with COVID to be in labor<br>☐ Other, please explain below<br>☐ not documented
Other	__________________________________
In the EHR documentation, what is the reported baby's sex([es] for those with multiple gestations)?	☐ Male<br>☐ Female<br>☐ Intersex<br>☐ Not documented
In the EHR documentation, How much did the baby 1 weigh at birth?<br>Pounds:Ounces____________	
In the EHR documentation, How much did the baby 2 weigh at birth?<br>Pounds:Ounces____________	
Pounds:	__________________________________
Ounces:	__________________________________
Did the baby have a birth defect (congenital anomaly)?	☐ Yes, it is documented in EHR<br>☐ No, it is not documented in EHR
In the EHR documentation, was the baby's defect detected before or after the patient's COVID diagnosis	☐ Before<br>☐ After<br>☐ not documented
In the EHR documentation, when was the baby's defect observed	☐ 1st<br>☐ 2nd<br>☐ 3rd<br>☐ at delivery<br>☐ 4th<br>☐ not documented
In the EHR documentation, what type of birth defect was the baby reported as having??	☐ Cardiac (heart)<br>☐ Lungs (pulmonary)<br>☐ Abdomen (sometimes called gastroschisis or omphalocele)<br>☐ Kidneys (renal)<br>☐ Bladder<br>☐ Limbs (extremities)<br>☐ Brain<br>☐ Face or lip (sometimes called cleft lip or palate)<br>☐ not documented
In the EHR documentation, was the baby reported being admitted to the neonatal intensive care unit (NICU)?	☐ Yes, it is documented in EHR<br>☐ No, it is not documented in EHR

In the EHR documentation, what is the zipcode where the baby was reported as being admitted to the NICU?	__________________________________
In the EHR documentation, is the baby delivered following your pregnancy with COVID reported as still living?	☐ Yes, it is documented in EHR<br>☐ No, it is not documented in EHR
In the EHR documentation, is it reported that the baby survived until they could be discharged home from the hospital after delivery?	☐ Yes, it is documented in EHR<br>☐ No, it is not documented in EHR

Table S1 Details of group assignment based on symptom documentation over time

Record ID	Group Status
1	Miscellaneous Excluded
3	Miscellaneous Excluded
4	Now
7	Symptoms Absent
8	Recovered
9	22 days but not now
11	Recovered
12	A year before only
13	Symptoms Absent
15	22 days till now
16	Miscellaneous Excluded
17	A year before only
18	A year before only
19	Symptoms Absent
20	A year before only
22	Symptoms Absent
23	Symptoms Absent
24	22 days till now
25	A year before only
26	22 days till now
28	Symptoms Absent
29	Miscellaneous Excluded
30	Symptoms Present
33	Miscellaneous Excluded
34	Miscellaneous Excluded
35	Symptoms Present
36	Symptoms Absent
38	A year before only
39	Symptoms Absent
40	Miscellaneous Excluded

Record ID	Group Status
41	Symptoms Present
42	Symptoms Absent
43	Symptoms Present
44	Symptoms Absent
45	Miscellaneous Excluded
47	Symptoms Absent
49	Miscellaneous Excluded
50	Symptoms Absent
51	Miscellaneous Excluded
52	A year before only
53	Symptoms Absent
54	Symptoms Absent
55	Symptoms Absent
56	A year before only
58	Symptoms Present
59	A year before only
60	Symptoms Present
61	Symptoms Present
63	Symptoms Present
64	A year before only
65	Symptoms Absent
66	Symptoms Present
67	Symptoms Absent
68	Symptoms Present
69	Miscellaneous Excluded
71	A year before only
72	Symptoms Absent
73	Miscellaneous Excluded
74	A year before only
75	Miscellaneous Excluded

Record ID	Group Status
76	Symptoms Present
77	Symptoms Absent
78	A year before only
79	A year before only
80	Miscellaneous Excluded
81	Symptoms Absent
82	Miscellaneous Excluded
83	Symptoms Absent
84	Symptoms Present
85	22 days till now
86	Symptoms Absent
87	Miscellaneous Excluded
88	Symptoms Absent
89	Symptoms Absent
90	Symptoms Absent
91	Symptoms Present
92	A year before only
93	Miscellaneous Excluded
94	Symptoms Absent
95	Miscellaneous Excluded
96	Symptoms Present
98	Symptoms Present
99	Symptoms Absent
100	Symptoms Present
101	Symptoms Present
102	Recovered
103	Miscellaneous Excluded
104	A year before only
105	Miscellaneous Excluded
106	Miscellaneous Excluded

Record ID	Group Status
107	Symptoms Present
108	Symptoms Present
109	22 days but not now
110	Recovered
111	Recovered
112	Symptoms Present
113	Symptoms Present
114	Symptoms Absent
115	Symptoms Present
119	Symptoms Absent
120	Miscellaneous Excluded
124	Symptoms Present
125	Recovered
129	Miscellaneous Excluded
130	Symptoms Absent
134	Symptoms Present
135	Miscellaneous Excluded
139	Miscellaneous Excluded
140	Symptoms Present
143	Symptoms Absent
145	Symptoms Present
148	Symptoms Present
150	Miscellaneous Excluded
153	Miscellaneous Excluded
156	Symptoms Present
159	Recovered
161	A year before only
164	Symptoms Present
166	Miscellaneous Excluded
169	A year before only

Record ID	Group Status
171	Symptoms Present
173	A year before only
176	Miscellaneous Excluded
179	A year before only
181	Symptoms Absent
185	A year before only
187	Symptoms Absent
190	A year before only
192	Symptoms Present
196	Recovered
201	Miscellaneous Excluded
202	Miscellaneous Excluded
207	Symptoms Present
208	Symptoms Present
212	Symptoms Present
213	Recovered
218	Symptoms Present
223	Miscellaneous Excluded
224	Symptoms Present
228	Symptoms Absent
229	Symptoms Present
233	Symptoms Absent
234	Miscellaneous Excluded
238	Symptoms Present
239	Miscellaneous Excluded
242	Miscellaneous Excluded
244	Symptoms Present
249	Symptoms Absent
378	Symptoms Present
